# Combined SHR and SIRI biomarkers predict increased coronary heart disease risk in type 2 diabetes

**DOI:** 10.17305/bb.2025.13032

**Published:** 2025-09-10

**Authors:** Zixuan Guo, Siqi Song, Hao Cheng, Changxu Xie, Meng Zhang, Mengyang Pei, Mengting Liu, Zican Shen

**Affiliations:** 1 Beijing Hospital of Traditional Chinese Medicine, Capital Medical University, Beijing, China; 2 Beijing Institute of Traditional Chinese Medicine, Beijing, China; 3 Department of Cardiology, Linquan County People’s Hospital, Fuyang, China; 4 Affiliated Hospital of Integrated Traditional Chinese and Western Medicine, Nanjing University of Chinese Medicine, Nanjing, China; 5 Department of Cardiology, Renmin Hospital of Wuhan University, Wuhan, China

**Keywords:** Stress hyperglycemia ratio, systemic inflammation response index, coronary heart disease, type 2 diabetes mellitus, risk assessment.

## Abstract

Coronary heart disease (CHD) is a leading cause of morbidity and mortality; patients with type 2 diabetes mellitus (T2DM) are at particularly high risk, highlighting the need for reliable biomarkers for early detection and risk stratification. We investigated whether combining the stress hyperglycemia ratio (SHR) and systemic inflammation response index (SIRI) improves CHD detection in T2DM. In this retrospective cohort of 943 T2DM patients undergoing coronary angiography, associations of SHR and SIRI with CHD were evaluated using multivariable logistic regression and restricted cubic splines; robustness was examined with subgroup and sensitivity analyses. Discriminative performance was assessed by receiver operating characteristic analysis and reclassification metrics (integrated discrimination improvement [IDI], net reclassification improvement [NRI]). Internal validation used bootstrapping, with calibration and discrimination yielding apparent and bias-corrected estimates. Of 943 patients, 600 had CHD. Multivariable models showed SHR (OR = 1.68; 95% CI, 1.14–2.46; *P* ═ 0.008) and SIRI (OR = 2.17; 95% CI, 1.54–3.05; *P* < 0.001) were independently associated with CHD, with nonlinear relationships (*P* for nonlinearity < 0.05). Findings were consistent across subgroups and sensitivity analyses. The combined SHR–SIRI model achieved an AUC of 0.813 (95% CI, 0.783–0.843), outperforming SHR alone (AUC = 0.773; 95% CI, 0.740–0.805) and SIRI alone (AUC = 0.745; 95% CI, 0.713–0.778), and significantly improved NRI and IDI (*P* < 0.05). All models showed strong discrimination and calibration. In conclusion, SHR and SIRI are independently associated with CHD in T2DM, and their combination enhances early identification of high-risk individuals.

## Introduction

Coronary heart disease (CHD) is globally acknowledged as one of the most prevalent and fatal health conditions. Despite significant advancements in interventional medical technology, CHD continues to pose a considerable burden on global public health security [1]. Diabetic patients exhibit a markedly higher risk of mortality following the onset of CHD compared to their non-diabetic counterparts [2]. Given the rising prevalence of diabetes, vigilant monitoring of this population is imperative [3].

Stress-induced hyperglycemia is a condition characterized by significantly elevated blood glucose levels resulting from various physiological or pathological stressors [4].

Elevated glucose levels at hospital admission serve as an independent predictor of CHD mortality among individuals with diabetes. However, this perspective does not account for the effects of chronic glycemic dysregulation, thereby imposing certain limitations [5, 6]. Roberts et al. [7] introduced the stress hyperglycemia ratio (SHR) to enhance the assessment of in-hospital blood glucose status. This metric is calculated by dividing admission fasting glucose by glycated hemoglobin. The SHR integrates both acute and chronic blood glucose parameters, effectively quantifying the extent of glycemic fluctuation and the body’s physiological response to stress [8].

Recent studies have revealed that in prediabetic and diabetic patients, the SHR is substantially correlated with the occurrence of CHD and multivessel coronary artery disease (CAD) [9]. Numerous studies indicate that high SHR independently predicts adverse cardiovascular outcomes and mortality in patients with type 2 diabetes mellitus (T2DM), acute myocardial infarction, or heart failure [10–12]. Furthermore, both acute and chronic dysglycemia hold significant prognostic value for major adverse cardiovascular events (MACEs) during short-term hospitalization and long-term outpatient follow-up. This is particularly evident in high-risk in-hospital settings, such as patients with myocardial infarction complicated by cardiogenic shock or those requiring advanced mechanical circulatory support in the intensive care unit [13, 14]. These findings underscore the clinical utility of SHR in cardiovascular risk assessment.

Systemic chronic inflammation is recognized as a significant contributor to vascular complications in diabetes mellitus. In patients with T2DM, the inflammatory response intensifies insulin resistance (IR) and endothelial cell dysfunction. This mechanism facilitates the uptake of excessive low-density lipoprotein (LDL) by monocytes, leading to accelerated foam cell formation and exacerbating the progression of atherosclerosis [15].

In recent years, the systemic inflammatory response index (SIRI) has garnered significant attention from medical practitioners. As a composite measure derived from neutrophil, lymphocyte, and monocyte counts, SIRI has emerged as a novel and reliable indicator of systemic inflammatory burden [16]. Recent studies have highlighted the prognostic significance of this marker across various cardiovascular conditions. It has been shown to predict mortality in heart failure patients with implantable devices and to assess the risk of contrast-induced nephropathy in individuals with non-ST-segment elevation myocardial infarction (NSTEMI) [17, 18]. Prior research has shown that a higher SIRI independently correlates with an increased risk of CHD among individuals with diabetes [19]. This finding aligns with a recent study by He et al. [20], which reported a significant association between SIRI and the severity of coronary vasculopathy. Furthermore, SIRI exhibits superior predictive performance for cardiovascular event-related mortality compared to traditional inflammatory markers [16, 19, 21].

Although both SHR and SIRI are independently associated with CHD, the potential interaction between SHR and SIRI in T2DM individuals remains undefined. Consequently, this research sought to evaluate the joint SHR and SIRI assessment for predicting CHD risk in T2DM patients. This study has substantial clinical implications for early detection of high-risk persons for CHD and improving the prognosis of T2DM patients.

## Materials and methods

### Study population

The present research utilized a retrospective analytical approach involving a total of 1096 patients with T2DM who underwent coronary angiography (CAG) at Linquan County People’s Hospital in Fuyang City from September 2023 to May 2025. The indications for CAG were established according to standard clinical guidelines [22] and included the following criteria: (1) typical or atypical chest pain with a high pre-test probability or abnormal results from non-invasive tests (e.g., stress ECG, stress echocardiography, and myocardial perfusion imaging); (2) non-ST-elevation acute coronary syndromes (NSTE-ACS); (3) ST-elevation myocardial infarction (STEMI); and (4) risk stratification prior to major non-cardiac surgery in high-risk patients. Results from CAG were used to classify participants into two groups: the T2DM group and the T2DM with CHD group.

**Figure 1.**
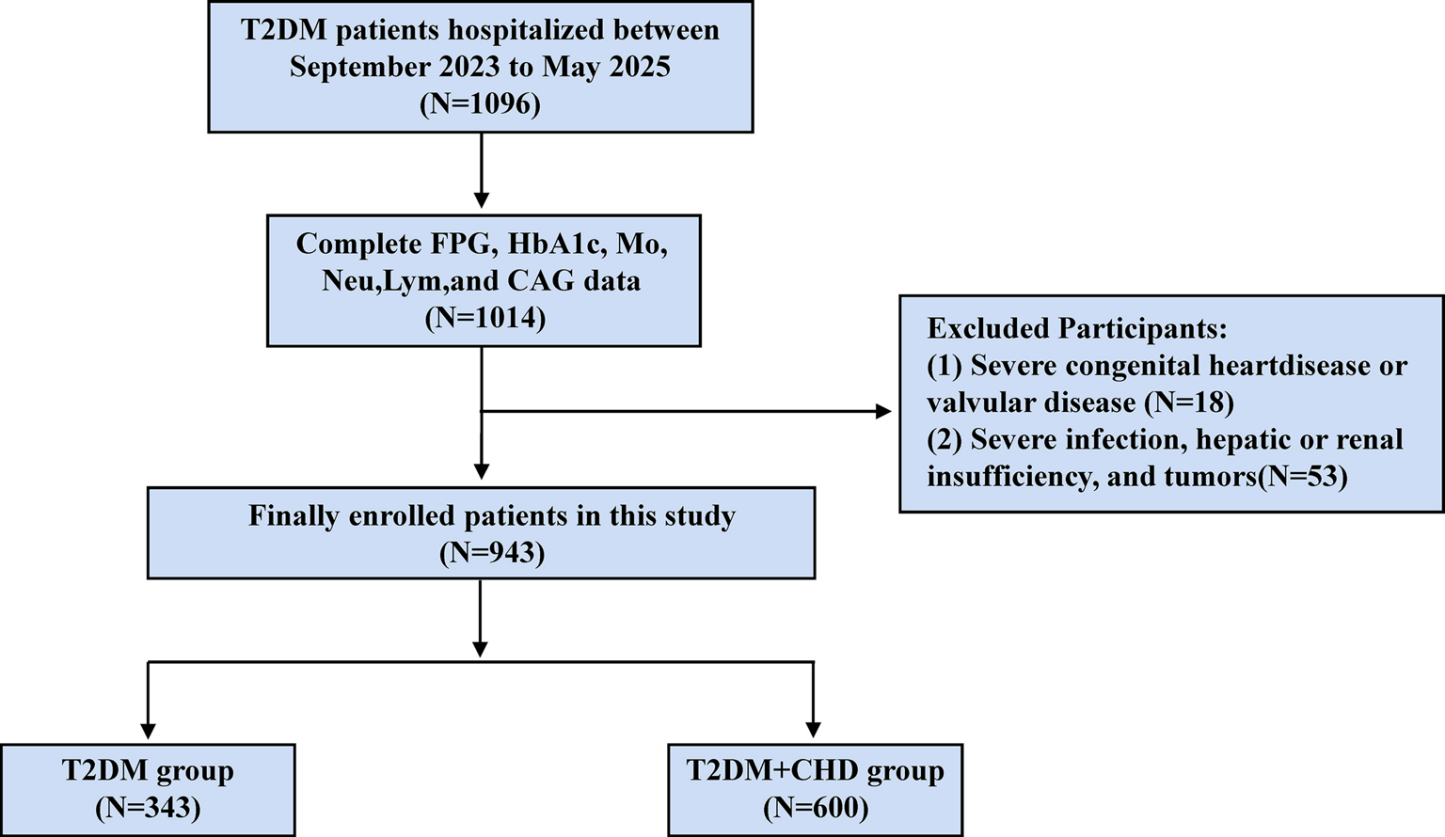
**Flowchart of patient recruitment.** Abbreviations: T2DM: Type 2 diabetes mellitus; FPG: Fasting plasma glucose; HbA1c: Glycosylated hemoglobin; Mo: Monocyte; Neu: Neutrophils; Lym: Lymphocyte; CAG: Coronary angiography; CHD: Coronary heart disease.

The following criteria were used to exclude patients from the study: (1) individuals diagnosed with severe valvular or congenital heart diseases; (2) individuals with significant hepatic dysfunction, defined as alanine aminotransferase levels at least five times the upper reference limit; (3) individuals with severely impaired renal function, indicated by an estimated glomerular filtration rate (eGFR) of less than 30 mL/min/1.73 m^2^; (4) individuals with severe infections, hematological disorders, autoimmune diseases, or malignancies; (5) individuals who experienced severe trauma or major surgery within the preceding three months; (6) individuals with incomplete CAG data; and (7) individuals lacking sufficient laboratory data to calculate SHR and SIRI. The patient enrollment flowchart (Figure 1) illustrates that 943 cases were included in the final analysis.

### Data collection

Two clinicians, blinded to the study’s purpose, retrieved baseline and laboratory hospitalization data for all participants from the inpatient electronic medical records. (1) Baseline information included: sex, age, admission body mass index (BMI), smoking status, admission systolic blood pressure (SBP), diastolic blood pressure (DBP), history of hypertension, heart failure, and the use of oral hypoglycemics, lipid-lowering agents, antihypertensives, and antiplatelet medications. (2) Laboratory measurements were obtained from fasting venous blood samples collected on the morning following hospital admission, after a standardized overnight fast of 8–12 h. These measurements included total cholesterol (TC), triglycerides (TG), LDL cholesterol (LDL-C), high-density lipoprotein cholesterol (HDL-C), fasting plasma glucose (FPG), glycated hemoglobin (HbA1c), serum creatinine (Scr), neutrophil count, lymphocyte count, and monocyte count. (3) CAG was performed by two cardiovascular intervention specialists, who were unaware of the study’s objectives, with detailed documentation of the outcomes. Inter-observer agreement for the assessment of ≥ 50% stenosis was excellent (Cohen’s κ ═ 0.90). In the event of initial discrepancies (*n* ═ 5), a consensus diagnosis was achieved with the involvement of a third senior interventional cardiologist as an arbiter.

### Laboratory methods

Fasting venous blood samples were collected in standardized tubes, processed within 2 h, and analyzed following stringent laboratory standard operating procedures. FPG levels were measured using the hexokinase method on a Roche Cobas c702 analyzer (Roche Diagnostics, Switzerland). HbA1c was quantified through high-performance liquid chromatography (HPLC) on a TOSOH HLC-723 G11 analyzer (TOSOH Corporation, Japan). A complete blood count was conducted using a Sysmex XN-3000 automated hematology analyzer (Sysmex Corporation, Kobe, Japan) with manufacturer-supplied reagents. Our laboratory adheres to rigorous internal quality control (IQC) and external quality assessment (EQA) protocols to ensure analytical accuracy and precision.

### Definitions

(1) The SHR and SIRI indices were calculated as follows: SHR = FPG (mmol/L) / (1.59 × HbA1c [%] – 2.59) [23]. This formula accounts for the non-linear relationship between HbA1c and average blood glucose levels, providing a more accurate assessment of acute hyperglycemia in the context of chronic glycemic exposure. SIRI is defined as (monocyte count × neutrophil count)/lymphocyte count [21]. (2) According to the American Diabetes Association criteria, the diagnosis of T2DM requires an FPG level of ≥7.0 mmol/L, an HbA1c of ≥6.5%, or a prior diagnosis of T2DM accompanied by ongoing glucose-lowering therapy [24]. (3) The diagnosis of CHD is established based on a ≥50% stenosis in one or more major coronary arteries, which include the left main, left anterior descending (LAD), left circumflex (LCx), or right coronary artery (RCA) [25].

### Ethical statement

This study was conducted in accordance with the Declaration of Helsinki and received ethical approval from the Ethics Committee of Linquan County People’s Hospital (No. LQXYLLMEC-2022-021). Due to its retrospective design, informed consent was not required. Furthermore, the confidentiality of all patient data was rigorously maintained.

### Statistical analysis

Continuous variables are reported as mean ± standard deviation for normal distributions or as median (interquartile range) for skewed distributions. The unpaired *t*-test was utilized for continuous variables with a normal distribution, while the Mann–Whitney *U* test was employed for non-normally distributed variables. Categorical variables, expressed in frequencies and percentages (%), were analyzed using the chi-square test or Fisher’s exact test. To examine associations between SHR, SIRI, and CHD, multiple logistic regression analysis was conducted. SHR and SIRI were categorized into tertiles and treated as categorical variables in the models. Model I represents the unadjusted analysis; Model II was adjusted for age, sex, and BMI; Model III included further adjustments for smoking status, admission SBP and DBP, history of hypertension and heart failure, and the use of hypoglycemic, lipid-lowering, antihypertensive, and antiplatelet medications. Restricted cubic splines (RCS) were employed to assess the nonlinear relationship between SHR or SIRI and CHD. Additionally, to validate the association between SHR/SIRI and CHD occurrence across diverse populations, subgroup analyses were performed based on age, gender, BMI, history of hypertension, and history of heart failure. The discriminative ability of SHR, SIRI, and their combination for predicting CHD was evaluated using receiver operating characteristic (ROC) curves.

Net reclassification improvement (NRI) and integrated discrimination improvement (IDI) were calculated to assess the added value of SHR and SIRI in predicting CHD. We conducted model diagnostics on the primary multivariate model (Model III) and performed internal validation using 1000 bootstrap iterations to obtain bias-corrected estimates of discriminative performance. Model calibration was evaluated with the Hosmer–Lemeshow test and visualized through a calibration plot. Additionally, the clinical utility of the prediction model was assessed using decision curve analysis (DCA), which quantifies the net benefit across a range of threshold probabilities. All statistical analyses were conducted using R software (version 4.3.0, R Foundation for Statistical Computing, Vienna, Austria). Statistical significance was defined as a two-tailed *P* value of less than 0.05.

## Results

### Clinical characteristics of T2DM and T2DM with CHD groups

This study enrolled 943 patients with T2DM, of whom 600 (63.6%) were diagnosed with CHD through CAG. The baseline demographics of the participants are presented in Table 1. Based on CAG findings, participants were categorized into two groups: a T2DM-only group (*n* ═ 343) and a T2DM with CHD group (*n* ═ 600). In the T2DM-only group, 41.1% were male, with a mean age of 70.9 ± 11.6 years; in the T2DM with CHD group, 45.2% were male, with a mean age of 72.0 ± 10.0 years. No significant differences were identified between the groups concerning age, gender, BMI, SBP, LDL-C, HDL-C, HbA1c, or history of hypertension (all *P* > 0.05). However, the prevalence of smokers, individuals with a history of heart failure, and those using hypoglycemic, lipid-lowering, antiplatelet, and antihypertensive medications was significantly higher in the T2DM with CHD group. Additionally, compared to the T2DM-only group, levels of TG, TC, FPG, Scr, SHR, and SIRI were significantly elevated in the T2DM with CHD group, while DBP was significantly lower (all *P* < 0.05).

**Table 1:** Demographic and clinical characteristics of included patients

**Characteristics**	**T2DM (*n* ═ 343)**	**T2DM+CHD (*n* ═ 600)**	***P***
*Demographics*			
Male (*n*,%)	141(41.1)	271(45.2)	0.254
Age (years)	70.9 ± 11.6	72.0 ± 10.0	0.131
BMI (kg/m^2^)	25.1 ± 5.0	25.1 ± 4.7	0.883
SBP (mmHg)	133.0 ± 26.3	131.0 ± 25.0	0.286
DBP (mmHg)	83.5 ± 15.5	81.2 ± 15.3	0.031
Smoking (*n*,%)	40(11.7)	122(20.3)	0.012
*Pastmedical history (*n*,%)*			
Hypertension	239(69.7)	431(71.8)	0.531
Heart failure	110(32.1)	389(64.8)	<0.001
Hypoglycemic drugs	274(79.9)	529(88.2)	<0.001
Dyslipidemia medications	218(63.6)	540(90.0)	<0.001
Antiplatelets drugs	117(34.1)	393(65.5)	<0.001
Antihypertensive drugs	205(59.8)	445(74.2)	<0.001
*Laboratory indicators*			
TG (mmol/L)	1.3(1.0–1.8)	1.4(1.0–2.1)	0.002
TC (mmol/L)	3.7 ± 1.2	3.9 ± 1.1	0.035
LDL-C (mmol/L)	2.0(1.5–2.6)	2.1(1.6–2.8)	0.103
HDL-C (mmol/L)	1.0 ± 0.3	0.9 ± 0.3	0.241
FPG (mmol/L)	6.9(5.5–8.9)	7.6(6.1–10.3)	<0.001
HbA1c (%)	7.7 ± 2.0	7.8 ± 1.7	0.892
Scr (µmol/L)	68.0(56.3–89.4)	78.6(61.5–112.2)	<0.001
SHR	0.7(0.6–0.9)	0.8(0.7–1.0)	0.002
SIRI	0.8 ± 0.4	1.0 ± 0.6	<0.001

Data are presented as means ± SDs, medians (interquartile ranges), or *n*(%). *P* values are descriptive and unadjusted for multiple comparisons. Formal inference is based on multivariable-adjusted models. Abbreviations: T2DM: Type 2 diabetes mellitus; CHD: Coronary heart disease; BMI: Body mass index; SBP: Systolic blood pressure; DBP: Diastolic blood pressure; TG: Triglyceride; TC: Total cholesterol; HDL-C: High-density lipoprotein cholesterol; LDL-C: Low-density lipoprotein cholesterol; FPG: Fasting plasma glucose; HbA1c: Glycosylated hemoglobin; Scr: Serum creatinine; SIRI: Systemic inflammation response index; SHR: Stress hyperglycemia ratio.

### Baseline characteristics stratified by SHR tertiles in T2DM patients

To investigate the relationship between varying levels of SHR and the incidence of CHD in patients with T2DM, participants were categorized into three subgroups based on SHR tertiles: T1 (*n* ═ 314; SHR ≤ 0.719), T2 (*n* ═ 314; 0.719 < SHR ≤ 0.918), and T3 (*n* ═ 315; SHR > 0.918). The median SHR values for these groups were 0.6, 0.8, and 1.1, respectively. As shown in Table 2, there were no significant differences among the three groups regarding age, gender, BMI, DBP, smoking prevalence, history of hypertension and heart failure, use of hypoglycemic agents, lipid-lowering drugs, antihypertensive medications, and antiplatelet therapies, as well as levels of LDL-C, HDL-C, and Scr levels (all *P* > 0.05). In comparison to the low-SHR tertile (T1), the high-SHR tertile (T3) demonstrated significantly elevated levels of SBP, TC, TG, FPG, HbA1c, and the SIRI. Additionally, the incidence of CHD was markedly higher in the T3 group compared to the T1 group (all *P* < 0.05).

**Table 2:** Baseline characteristics of study population according to tertiles of SHR

**Characteristics**	**T1 (*n* ═ 314)**	**T2 (*n* ═ 314)**	**T3 (*n* ═ 315)**	***P***
*Demographics*				
Male (*n*,%)	149(47.5)	135(43.0)	128(40.6)	0.242
Age (years)	71.5 ± 10.3	72.0 ± 9.9	71.6 ± 10.6	0.971
BMI (kg/m^2^)	25.3 ± 4.5	25.2 ± 4.3	24.8 ± 5.5	0.388
SBP (mmHg)	129.0 ± 25.5	132.0 ± 24.6	134.0 ± 26.1	0.030
DBP (mmHg)	80.5 ± 14.8	82.2 ± 14.7	83.4 ± 16.6	0.084
Smoking (*n*,%)	49(15.6)	60(19.1)	53(16.8)	0.413
*Pastmedical history (*n*,%)*				
Hypertension	213(67.8)	233(74.2)	224(71.1)	0.189
Heart failure	157(50.0)	161(51.3)	181(57.5)	0.114
Hypoglycemic drugs	264(84.1)	267(85.0)	272(86.3)	0.609
Dyslipidemia medications	245(78.0)	254(80.9)	259(82.2)	0.319
Antiplatelets drugs	168(53.5)	161(51.3)	181(57.5)	0.263
Antihypertensive drugs	203(64.6)	226(72.0)	221(70.2)	0.099
*Laboratory indicators*				
TG (mmol/L)	1.2(0.9–1.7)	1.3(1.0–1.9)	1.4(1.0–2.0)	0.028
TC (mmol/L)	3.6 ± 1.1	3.8 ± 1.1	3.9 ± 1.2	0.004
LDL-C (mmol/L)	1.9(1.4–2.6)	2.1(1.5–2.7)	2.2(1.5–2.9)	0.107
HDL-C (mmol/L)	0.9 ± 0.3	0.9 ± 0.2	1.0 ± 0.3	0.411
FPG (mmol/L)	5.8(4.7–7.0)	7.2(6.1–8.4)	10.3(8.2–13.4)	<0.001
HbA1c (%)	7.4 ± 1.5	7.5 ± 1.7	8.3 ± 2.0	<0.001
Scr (µmol/L)	73.7(58.9–104.8)	71.4(59.3–97.5)	78.5(59.4–104.6)	0.097
SHR	0.6(0.5–0.7)	0.8(0.7–0.9)	1.1(1.0–1.3)	<0.001
SIRI	0.8 ± 0.4	0.9 ± 0.4	1.1 ± 0.7	<0.001
CHD (*n*,%)	178(56.7)	207(65.9)	215(68.3)	0.005

Data are presented as means ± SDs, medians (interquartile ranges), or *n*(%). The groups were stratified by the tertiles of the SHR (T1, SHR ≤ 0.719; T2, 0.719 < SHR ≤ 0.918; T3, SHR > 0.918). *P* values are descriptive and unadjusted for multiple comparisons. Formal inference is based on multivariable-adjusted models. Abbreviations: T2DM: Type 2 diabetes mellitus; CHD: Coronary heart disease; BMI: Body mass index; SBP: Systolic blood pressure; DBP: Diastolic blood pressure; TG: Triglyceride; TC: Total cholesterol; HDL-C: High-density lipoprotein cholesterol; LDL-C: Low-density lipoprotein cholesterol; FPG: Fasting plasma glucose; HbA1c: Glycosylated hemoglobin; Scr: Serum creatinine; SIRI: Systemic inflammation response index; SHR: Stress hyperglycemia ratio.

### The relationship between SHR, SIRI, and CHD

Three logistic regression models were developed to examine the associations among SHR, SIRI, and CHD. Model I was unadjusted, while Model II was adjusted for gender, age, and BMI; Model III included further adjustments for smoking status, SBP, DBP, histories of hypertension, heart failure, and the use of hypoglycemic, lipid-lowering, antihypertensive, and antiplatelet medications, building on Model II. To assess potential multicollinearity between SHR and SIRI—both of which are potential markers of systemic stress—the variance inflation factor (VIF) was calculated for all variables in the fully adjusted Model III. The VIF values for all variables were less than 2, well below the common threshold of 5–10, indicating that multicollinearity was not a significant concern and would not distort the regression estimates or their interpretations.

**Table 3:** The association between SHR and CHD

**SHR**	**Model I**		**Model II**		**Model III**	
	**OR (95% CI)**	***P***	**OR (95% CI)**	***P***	**OR (95% CI)**	***P***
T1	Ref.		Ref.		Ref.	
T2	1.49(1.08–2.06)	0.016	1.50(1.09–2.08)	0.014	1.66(1.13–2.43)	0.009
T3	1.67(1.21–2.32)	0.002	1.71(1.23–2.38)	0.001	1.68(1.14–2.46)	0.008

Model I: Unadjusted; Model II: Adjusted for age, sex, and BMI; Model III: Model II+ admission history of SBP, DBP, smoking history, hypertension, heart failure, antihypertensive, dyslipidemia, hypoglycemic, and antiplatelet medication history. The tertiles of the SHR (T1, SHR ≤ 0.719; T2, 0.719 < SHR ≤ 0.918; T3, SHR > 0.918). Abbreviations: SHR: Stress hyperglycemia ratio; SBP: Systolic blood pressure; DBP: Diastolic blood pressure; OR: Odds ratio; CI: Confidence interval; Ref: Reference; CHD: Coronary heart disease; BMI: Body mass index.

As presented in Table 3, SHR was stratified into tertiles (T1–T3), with the first tertile (T1) serving as the reference category. In Model I, the T3 group exhibited a significantly higher risk of CHD compared to the T1 group, with an odds ratio (OR) of 1.67 (95% confidence interval [CI]: 1.21–2.32; *P* ═ 0.002). This association remained consistent and significant across subsequent models: in Model II, the OR was 1.71 (95% CI: 1.23–2.38; *P* ═ 0.001), and in the fully adjusted Model III, the OR was 1.68 (95% CI: 1.14–2.46; *P* ═ 0.008). Similarly, the association between SIRI and CHD was assessed across the three logistic regression models. As indicated in Table 4, the highest tertile (T3) of SIRI was consistently associated with an increased risk of CHD compared to the reference tertile (T1), with an OR of 2.24 (95% CI: 1.61–3.13; *P* < 0.001) in Model I, 2.14 (95% CI: 1.53–3.01; *P* < 0.001) in Model II, and 2.17 (95% CI: 1.54–3.05; *P* < 0.001) in the fully adjusted Model III.

**Table 4:** The association between SIRI and CHD

**SIRI**	**Model I**		**Model II**		**Model III**	
	**OR (95% CI)**	***P***	**OR (95% CI)**	***P***	**OR (95% CI)**	***P***
T1	Ref.		Ref.		Ref.	
T2	1.44(1.05–1.98)	0.026	1.41(1.02–1.95)	0.036	1.43(1.04–1.99)	0.026
T3	2.24(1.61–3.13)	<0.001	2.14(1.53–3.01)	<0.001	2.17(1.54–3.05)	<0.001

Model I: Unadjusted; Model II: Adjusted for age, sex, and BMI; Model III: Model II+ admission history of SBP, DBP, smoking history, hypertension, heart failure, antihypertensive, dyslipidemia, hypoglycemic, and antiplatelet medication history. The tertiles of the SIRI (T1, SIRI ≤ 0.678; T2, 0.678 < SIRI ≤ 0.978; T3, SIRI > 0.978). Abbreviations: SIRI: Systemic inflammation response index; SBP: Systolic blood pressure; DBP: Diastolic blood pressure; OR: Odds ratio; CI: Confidence interval; Ref: Reference; CHD: Coronary heart disease; BMI: Body mass index.

**Figure 2.**
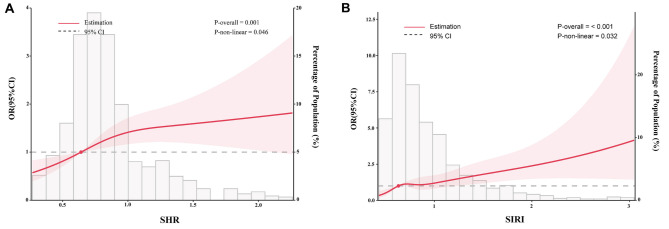
**Restricted cubic spline curves for the association of SHR (A) and SIRI (B) with the risk of CHD.** SHR and SIRI were analyzed as continuous variables using restricted cubic splines. The curves demonstrate significant nonlinear dose–response relationships with CHD risk (*P* for nonlinearity < 0.05), supporting the tertile-based categorization used for clinical interpretation. Abbreviations: SHR: Stress hyperglycemia ratio; SIRI: Systemic inflammation response index; OR: Odds ratio; CI: Confidence interval; CHD: Coronary heart disease.

To facilitate clinical interpretation, SHR and SIRI were categorized into tertiles; however, RCS confirmed non-linear associations with CHD (Figure 2). When analyzed as continuous variables using RCS, both biomarkers demonstrated significant non-linear dose–response relationships with CHD risk (P for non-linearity < 0.05), thereby validating the tertile-based approach. Additionally, we conducted two sensitivity analyses. First, after adjusting for TC, TG, LDL, and HDL, the results indicated that SHR and SIRI remained independent predictors of CHD. Further adjustments for eGFR were performed, yielding results consistent with the primary model and confirming the stability of the study findings (Table S1).

### Subgroup analysis

**Figure 3.**
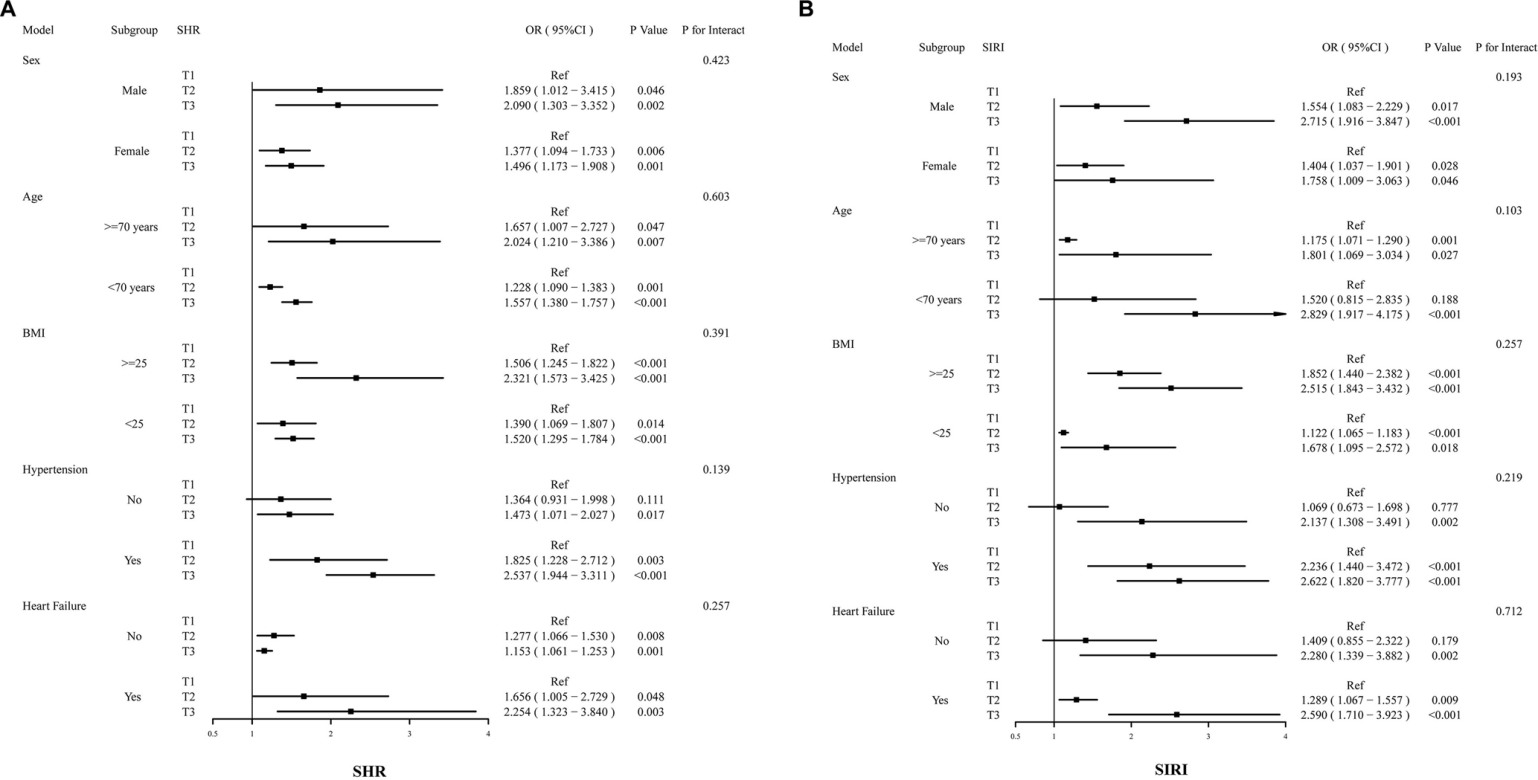
**Forest plots for subgroup analyses of SHR (A) and SIRI (B) with CHD.** Forest plots show multivariable-adjusted ORs with 95% CIs for CHD across tertiles (T1 = reference) within prespecified strata: sex (male/female), age (≥70 vs <70 years), BMI (≥25 vs <25 kg/m^2^), hypertension (yes/no), and heart failure (yes/no). *P* values refer to within-subgroup associations; “*P* for interaction” tests heterogeneity across strata. Results were consistent across all subgroups with no significant effect modification (all *P* for interaction > 0.05), supporting the stability of the findings. Abbreviations: SHR: Stress hyperglycemia ratio; SIRI: Systemic inflammation response index; CHD: Coronary heart disease; OR: Odds ratio; CI: Confidence interval; Ref: Reference; BMI: Body mass index.

We conducted subgroup analyses of SHR and SIRI to assess their impact on the occurrence of CHD across various demographic groups. Using the T1 group as a reference, our results indicated no significant interactions across subgroups categorized by age, gender, BMI, hypertensive status, and history of heart failure, thereby reinforcing the robustness of our findings (all interaction *P* > 0.05, Figure 3). This study confirms that elevated SHR and SIRI are significant predictors of CHD in individuals with T2DM.

**Table 5:** ROC curves analysis of SHR or/and SIRI to predict CHD

**Variable**	**AUC**	**95% CI**	***P***	**Sensitivity**	**Specificity**	**Youden’s index**	**Cut-off**
SHR	0.773	0.740–0.805	<0.001	0.701	0.746	0.446	0.839
SIRI	0.745	0.713–0.778	<0.001	0.700	0.646	0.347	0.476
SHR+SIRI	0.813	0.783–0.843	<0.001	0.815	0.711	0.526	0.560

Abbreviations: ROC: Receiver operating characteristic; AUC: Area under the curve; CI: Confidence interval; SIRI: Systemic inflammation response index; SHR: Stress hyperglycemia ratio; CHD: Coronary heart disease.

### Predictive value of SHR and SIRI for CHD

**Figure 4.**
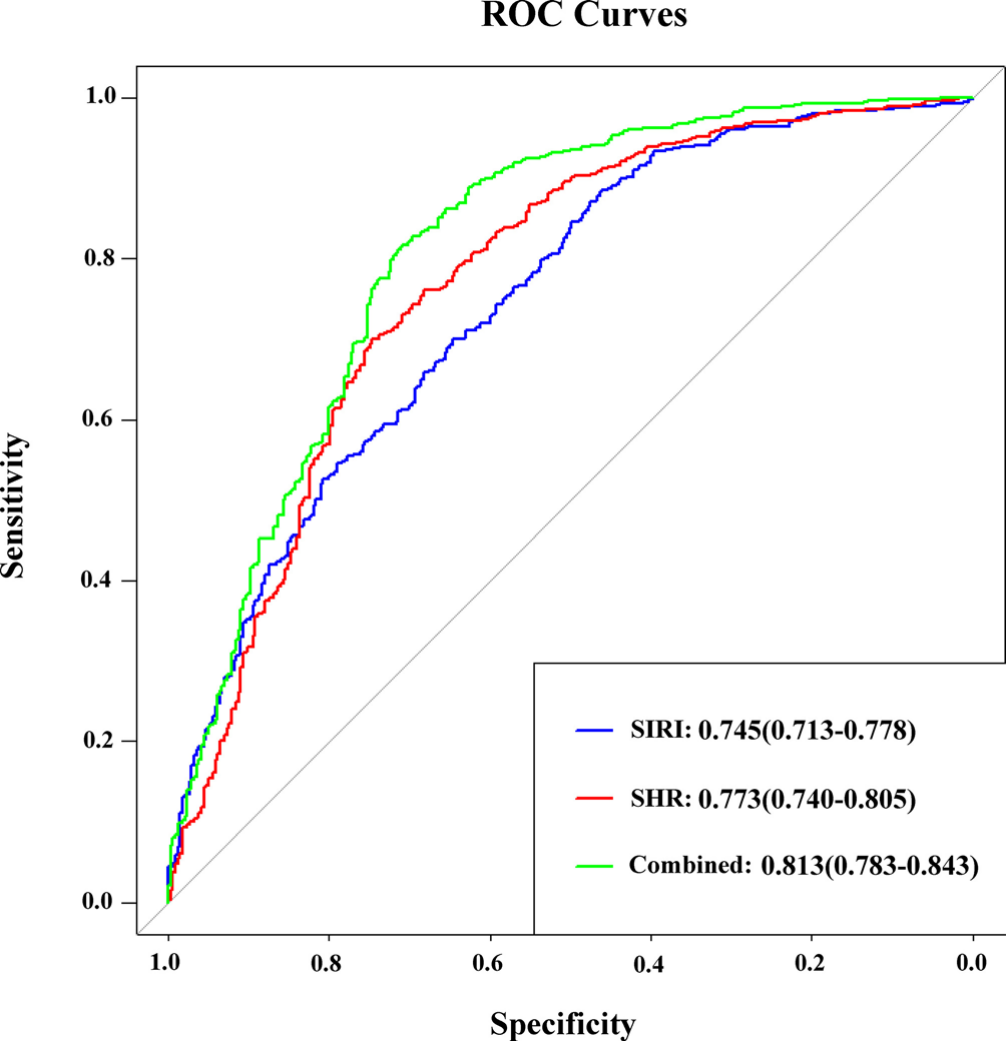
**Receiver operating characteristic curves of SHR, SIRI, and their combination in predicting CHD.** AUCs (95% CI) were 0.773 (0.740–0.805) for SHR (red), 0.745 (0.713–0.778) for SIRI (blue), and 0.813 (0.783–0.843) for the combined model (green). Corresponding sensitivities/specificities were 70.1%/74.6% (SHR), 70.0%/64.7% (SIRI), and 81.5%/71.1% (combined), indicating superior discrimination of the combined model. Abbreviations: SHR: Stress hyperglycemia ratio; SIRI: Systemic inflammation response index; CHD: Coronary heart disease.

We conducted ROC curve analysis to assess the predictive effects of SHR, SIRI, and their combination on the incidence of CHD in patients with T2DM. As shown in Table 5 and Figure 4, the AUC for SHR and SIRI were 0.773 (95% CI: 0.740–0.805) and 0.745 (95% CI: 0.713–0.778), respectively. The sensitivity values for SHR and SIRI were 70.1% and 70.0%, respectively, while their specificities were 74.6% and 64.7%. The AUC for the combination of SHR and SIRI was 0.813 (95% CI: 0.783–0.843), with a sensitivity of 81.5% and a specificity of 71.1%. These results indicate that the combination of SHR and SIRI offers superior predictive capability for CHD incidence compared to each indicator used individually. This finding supports the use of this combined indicator for enhanced risk assessment of CHD in patients with T2DM.

To further validate the effectiveness of the composite indicators, we conducted analyses of the NRI and IDI. The combination of SHR and SIRI significantly enhanced predictive ability compared to SHR alone, resulting in an NRI increase of 0.335 (95% CI: 0.211–0.460) and an IDI increase of 0.050 (95% CI: 0.037–0.062). When compared to SIRI alone, this combination similarly improved prediction, yielding NRI and IDI increases of 0.422 (95% CI: 0.295–0.549) and 0.039 (95% CI: 0.027–0.050), respectively; all differences were statistically significant (*P* < 0.05, Table S2). The combined indicators demonstrated a significant overall improvement in predictive accuracy compared to SHR or SIRI used individually. These findings advocate for the routine consideration of both SHR and SIRI in clinical management of T2DM, as their combined application provides a more efficient quantitative approach for managing CHD risk in this patient population.

### Model calibration

To obtain more robust and realistic performance estimates, we conducted internal validation utilizing bootstrapping with 1000 resamples. In this internal bootstrap validation, the combined model incorporating both SHR and SIRI exhibited superior discriminatory performance, achieving a bias-corrected AUC of 0.846 (Figure S1 and Table S3). This value was significantly higher than the AUCs for SHR (0.793) and SIRI (0.742) when analyzed individually. Furthermore, as displayed in Table S4, the combined model demonstrated significant improvements in reclassification metrics compared to each biomarker alone. Specifically, relative to SHR, the combined model increased the NRI by 0.403 (95% CI: 0.236–0.571) and the IDI by 0.053 (95% CI: 0.033–0.073). Similarly, when compared to SIRI alone, it increased the NRI by 0.424 (95% CI: 0.257–0.591) and the IDI by 0.059 (95% CI: 0.037–0.080). The calibration of the multivariable logistic regression model (Model III) was evaluated using the Hosmer–Lemeshow test. The results indicated that both the internal and validation models exhibited non-significant Hosmer–Lemeshow test results (*P* > 0.05), suggesting no statistically significant deviation between predicted and observed probabilities (Figure S2). These findings confirm the robust discriminative performance of the combined model.

Finally, DCA was employed to assess the clinical utility of the combined model (Figure S3). The *y*-axis represents the net benefit, while the *x*-axis denotes the threshold probability. Each model’s clinical utility is illustrated by a distinct curve. In both the original dataset and the internal bootstrap validation, the model demonstrated a positive net benefit across a threshold probability range of approximately 10%–70%. This suggests its potential clinical utility for guiding decision-making across a wide spectrum of risk thresholds, indicating that utilizing the model for risk stratification offers a significant advantage compared to disregarding it.

## Discussion

This research provides the first comprehensive quantification of the synergistic influence of SHR and SIRI on the susceptibility to CHD in diabetic patients. The multidimensional validation of their joint prognostic utility introduces a novel biomarker pair for the early screening of cardiovascular complications. The principal conclusions are as follows: (1) elevated levels of SHR and SIRI are significantly associated with an increased risk of CHD in patients with T2DM; (2) SHR and SIRI demonstrate a nonlinear relationship with the incidence of CHD; and (3) the combined use of SHR and SIRI markedly enhances discriminative performance compared to individual indicators, facilitating more precise CHD risk stratification for T2DM patients.

Clinically referred to as stress hyperglycemia, this transient glycemic elevation results from neuroendocrine dysfunction and cytokine storms, often observed during severe disease states [26]. In patients with T2DM, acute hyperglycemia exacerbated by neuroendocrine dysfunction impairs glucose transporter function in cardiomyocytes, worsening IR [27]. This acute hyperglycemic state induces significant oxidative stress and inflammatory responses, including the generation of reactive oxygen species. These processes enhance coagulation, diminish fibrinolytic activity through increased platelet aggregation and decreased fibrinolysis, promote myocardial injury, and expand the infarct area, consequently elevating mortality risk [28–30].

Hyperglycemia contributes to coronary artery calcification and left ventricular remodeling by impairing vascular endothelial cell function, specifically through reduced nitric oxide bioavailability. This dysfunction further increases the risk of CHD mortality [31]. In contrast to random blood glucose measurements taken at admission, the use of SHR as a novel biomarker provides a more accurate reflection of acute hyperglycemia. SHR corrects for the influence of chronic blood glucose levels, particularly in patients with T2DM who exhibit chronic glucose metabolism abnormalities. A nationwide cohort study identified SHR as an independent predictor of one-year all-cause mortality among patients with STEMI and diabetes [32].

Previous research consistently associates the SHR with increased rates of cardiovascular morbidity and mortality among diabetic inpatients in hospital settings, confirming its status as an independent marker of poor clinical outcomes [33–35]. Furthermore, a retrospective study by Zhang et al. [9] revealed that a high SHR is closely linked to an elevated risk of CHD in diabetic patients and is significantly associated with the severity of coronary artery lesions. This study, which enrolled 943 patients with T2DM who underwent CAG, found a strong positive correlation between elevated SHR and CHD risk in this population. After adjusting for clinical cardiovascular risk factors, the association remained statistically significant (Table 3), and results were consistent across all subgroup analyses (Figure 3).

Although our analyses accounted for multiple confounders and demonstrated a significant association, the cross-sectional design limits our ability to fully address the issue of reverse causality. It is plausible that the stress associated with symptomatic CAD could elevate SHR values, rather than SHR being solely a predisposing risk factor. Nonetheless, the biological mechanisms through which acute hyperglycemia exacerbates oxidative stress, inflammation, and endothelial dysfunction provide a compelling rationale for SHR’s contributory role in the pathogenesis and progression of CAD [30].

Therefore, SHR should be regarded as an integrative risk marker that reflects both the acute stress associated with prevalent disease and an underlying susceptibility to glycemic dysregulation, which may contribute to the progression of coronary atherosclerosis. Future prospective studies are essential to clarify this temporal relationship and definitively establish SHR as a predictive risk factor for incident CAD events in populations with T2DM.

In addition to the effects of acute hyperglycemia, chronic inflammatory responses are closely linked to the progression of CHD, particularly in patients with T2DM. Chronic inflammation exacerbates IR by activating a cascade of inflammatory mediators, intensifying oxidative stress within the vascular wall, disrupting normal lipid metabolism, and impairing endothelial cell function [36]. In recent years, the SIRI has garnered significant attention as a definitive indicator of chronic inflammation. Thus, SHR and SIRI likely share common underlying pathophysiological mechanisms. Stress-induced hyperglycemia can activate pro-inflammatory cytokines and increase oxidative stress, further exacerbating endothelial dysfunction and heightening atherosclerotic plaque instability. Conversely, chronic inflammation may impair insulin signaling and promote hyperglycemia, creating a self-perpetuating cycle. This bidirectional relationship provides a mechanistic basis for the combined use of SHR and SIRI in predicting CHD risk in patients with T2DM, a finding corroborated by a study conducted by Yan et al. [37].

Although the SIRI and the SHR may be physiologically interrelated, statistical testing for multicollinearity (VIF < 2.0) confirmed that they offer independent informational value within our predictive model. Elevated SIRI indicates heightened systemic inflammation, characterized by increased neutrophil and monocyte counts alongside decreased lymphocyte counts, reflecting a state of immune dysregulation. Activated neutrophils release proteases and oxidants; monocytes and macrophages contribute to foam cell formation and plaque destabilization; and lymphopenia indicates elevated physiological stress, which is associated with a poorer prognosis. This immune–inflammatory environment facilitates metabolic disorders and atherosclerosis, thereby driving the progression of CHD [38]. Lin et al. previously demonstrated that SIRI is a more robust marker of systemic inflammatory response than high-sensitivity C-reactive protein (hs-CRP). In their study, diabetic patients showed that higher SIRI levels correlated with an increased risk of cardiovascular complications, including CHD [19]. Additionally, a recent prospective study indicates that SIRI exhibits enhanced predictive capability for cardiovascular mortality compared to other inflammatory markers [16]. Similarly, our study demonstrated that SIRI is independently associated with the manifestation of CHD among patients with T2DM; this correlation persisted after adjusting for relevant confounders (Table 4). Furthermore, the results of the subgroup analysis were consistent, confirming the robustness of our study’s conclusions (Figure 3). These findings establish a foundation for the early identification of high-risk CHD populations using SIRI and prompt therapeutic intervention.

SHR and SIRI are closely associated with the onset and progression of IR and CHD. This research represents the first investigation into the discriminative performance of combining SHR and SIRI to predict CHD in patients with T2DM. ROC curve analysis indicated that the AUC for SHR in predicting CHD risk among T2DM patients was 0.772, compared to 0.745 for SIRI. The combined AUC of both predictors increased to 0.813 (see Table 5 and Figure 4). This finding suggests that the predictive value of the combined SHR and SIRI is significantly superior to that of individual markers, a conclusion supported by NRI and IDI analyses (see Table S2). Furthermore, the robustness of this finding was confirmed through internal bootstrap validation, employing sampling with replacement across 1000 iterations (see Table S3 and S4).

These results underscore the clinical importance of effective glycemic control and cardiovascular risk assessment in T2DM populations. For patients with T2DM, it is crucial to maintain both glycemic and inflammatory levels within a relatively normal range while avoiding hypoglycemia. Persistent severe stress-induced hyperglycemia and chronic inflammation can synergistically exacerbate CHD progression through mechanisms such as oxidative stress, vascular endothelial damage, and atherosclerosis. Addressing these pathological factors can significantly reduce the incidence of major cardiovascular events.

Establishing clinically relevant cut-off values is essential for the practical application of novel biomarkers. Based on our ROC analysis, we propose cut-off values of 0.84 for SHR and 0.48 for SIRI for initial risk stratification in T2DM patients. These values align with those reported in previous studies on cardiovascular and metabolic diseases [39–41]. Additionally, the cut-off values identified in our study—specifically for T2DM patients at risk for CHD—may reflect the unique interaction between chronic glucose dysregulation and inflammation characteristic of this population. An integrative assessment of SHR and SIRI may enable clinicians to more accurately identify high-risk patients for CHD and provide more reliable decision-making support for early intervention.

Moreover, these biomarkers can complement traditional risk factors, ultimately aiming to improve cardiovascular outcomes in individuals with T2DM. Future prospective studies are necessary to validate these cut-off values and evaluate the clinical efficacy of this biomarker-guided approach. This research marks the inaugural confirmation that the integrated evaluation of SHR and SIRI significantly enhances the predictive accuracy of CHD risk in T2DM patients. This finding offers a more precise risk stratification tool for clinical practice, facilitating a transition from single-indicator management to a comprehensive multi-indicator management paradigm, thereby having meaningful implications for optimizing cardiovascular outcomes in T2DM patients.

### Strengths and limitations

The primary strength of this research lies in its substantial population of chronic T2DM patients, yielding clinically significant results. This study is the first to examine the discriminative performance of combining the SHR and the SIRI in relation to the occurrence of CHD. However, the research has notable limitations. First, due to the inherent constraints of retrospective study designs, it is impossible to establish causality. Second, while we adjusted for a comprehensive array of clinically relevant covariates, our study lacked data on several key prognostic variables, specifically diabetes duration, baseline eGFR, and albuminuria. We recognize that the absence of these variables may introduce residual confounding and limit the comprehensiveness of our risk model. Further studies that incorporate these parameters are necessary to validate our findings. Lastly, as a single-center retrospective study, our dataset was confined to patients referred for CAG, a group that likely exhibits more severe symptoms or a higher pre-test probability of CHD. This referral bias restricts the generalizability of our results to the broader asymptomatic T2DM population. Therefore, future multi-center prospective studies that include community-based asymptomatic cohorts are essential for validating these findings. Such research would enhance the clinical translation of the joint evaluation of SHR and SIRI from associative evidence to causal intervention applications.

## Conclusion

SHR and SIRI exhibit a significant correlation with the onset of CHD in patients with T2DM. These metrics may serve as complementary factors alongside traditional cardiovascular risk indicators. The integrated evaluation of SHR and SIRI enhances CHD risk stratification in T2DM patients, facilitating more precise early identification of high-risk individuals and ultimately improving patient outcomes.

## Supplemental data

Supplemental data are available at the following link: https://www.bjbms.org/ojs/index.php/bjbms/article/view/13032/4002.

## Data Availability

The data produced and examined in this study are not publicly available due to privacy and ethical considerations. However, they are available from the corresponding author upon justified and reasonable request.
